# Budding Yeast Greatwall and Endosulfines Control Activity and Spatial Regulation of PP2A^Cdc55^ for Timely Mitotic Progression

**DOI:** 10.1371/journal.pgen.1003575

**Published:** 2013-07-04

**Authors:** Maria Angeles Juanes, Rita Khoueiry, Thomas Kupka, Anna Castro, Ingrid Mudrak, Egon Ogris, Thierry Lorca, Simonetta Piatti

**Affiliations:** 1 Centre de Recherche en Biochimie Macromoléculaire, Montpellier, France; 2 Max F. Perutz Laboratories, Medical University of Vienna, Vienna, Austria; Cancer Research UK London Research Institute, United Kingdom

## Abstract

Entry into mitosis is triggered by cyclinB/Cdk1, whose activity is abruptly raised by a positive feedback loop. The Greatwall kinase phosphorylates proteins of the endosulfine family and allows them to bind and inhibit the main Cdk1-counteracting PP2A-B55 phosphatase, thereby promoting mitotic entry. In contrast to most eukaryotic systems, Cdc14 is the main Cdk1-antagonizing phosphatase in budding yeast, while the PP2A^Cdc55^ phosphatase promotes, instead of preventing, mitotic entry by participating to the positive feedback loop of Cdk1 activation. Here we show that budding yeast endosulfines (Igo1 and Igo2) bind to PP2A^Cdc55^ in a cell cycle-regulated manner upon Greatwall (Rim15)-dependent phosphorylation. Phosphorylated Igo1 inhibits PP2A^Cdc55^ activity *in vitro* and induces mitotic entry in *Xenopus* egg extracts, indicating that it bears a conserved PP2A-binding and -inhibitory activity. Surprisingly, deletion of *IGO1* and *IGO2* in yeast cells leads to a decrease in PP2A phosphatase activity, suggesting that endosulfines act also as positive regulators of PP2A in yeast. Consistently, *RIM15* and *IGO1/2* promote, like PP2A^Cdc55^, timely entry into mitosis under temperature-stress, owing to the accumulation of Tyr-phosphorylated Cdk1. In addition, they contribute to the nuclear export of PP2A^Cdc55^, which has recently been proposed to promote mitotic entry. Altogether, our data indicate that Igo proteins participate in the positive feedback loop for Cdk1 activation. We conclude that Greatwall, endosulfines, and PP2A are part of a regulatory module that has been conserved during evolution irrespective of PP2A function in the control of mitosis. However, this conserved module is adapted to account for differences in the regulation of mitotic entry in different organisms.

## Author Summary

In all eukaryotic cells chromosome partition during mitosis requires a number of processes, including the formation of the mitotic spindle, i.e. the machinery that drives chromosome segregation to the daughter cells. Mitotic entry requires a delicate balance between protein phosphorylation, driven by cyclin-dependent kinases (CDKs), and protein dephosphorylation, carried out by specific phosphatases that counteract CDK activity. A critical threshold in CDK activity is indeed required for mitotic entry. In the past few years the Greatwall kinase has also been implicated in mitotic entry through phosphorylation of proteins of the endosulfine family, which in turn inhibit the activity of the PP2A phosphatase that would otherwise dephosphorylate CDK targets. Whether Greatwall and endosulfines have a mitotic function in budding yeast, where PP2A promotes, rather than inhibits, mitotic entry has not been established. Here we show that the Greatwall-endosulfine-PP2A regulatory module is conserved also in budding yeast and that endosulfines from different species are interchangeable for their mitotic function. However, in budding yeast cells endosulfines contribute to full activation and proper localization of PP2A, suggesting that they act as both inhibitors and activators of PP2A. Our data emphasize how the same regulatory module is adapted to meet specific mitotic features in different organisms.

## Introduction

Entry into mitosis in eukaryotic cells is driven by cyclin-dependent kinases (CDKs) bound to B-type cyclins. Many targets of cyclinB/CDKs have been identified in different organisms and include proteins involved in mitotic spindle formation/elongation, nuclear envelope breakdown, chromosome condensation and segregation.

Several mechanisms contribute to the rapid raise in cyclinB/CDKs activity at the onset of mitosis: i) cyclin accumulation, through transcriptional activation and inhibition of their proteolysis; ii) phosphorylation of their catalytic subunit Cdk1 by Cdk1-activating kinases (CAKs); iii) removal of Cdk1 inhibitory phosphorylations on Thr14 and Tyr15 (reviewed in [1]. Phosphorylation on Thr14 and Tyr15 of Cdk1 is carried out by Wee1 (Swe1 in budding yeast) and Myt1 protein kinases and is reversed by Cdc25-like phosphatases (Mih1 in budding yeast) that promote entry into mitosis. Polo kinase, which is activated in mitosis by cyclinB/Cdk1 [2], [3], activates in turn Cdc25 [4], [5]. In addition, the Wee1 kinase is phosphorylated and downregulated by cyclinB/Cdk1 [6], [7], [8], [9]. Together, these data have led to the idea of a positive autoregulatory loop for cyclinB/Cdk1 activation at the onset of M phase [10], [11]. In budding yeast a positive feedback loop for mitotic CDKs also exists, but differs from that of other organisms in many respects. The mitotic CDKs Clb1-4/Cdk1 drive a mitotic transcriptional program that leads to accumulation of several mitotic proteins, including the Clb1-4 cyclins themselves and the polo kinase Cdc5 (reviewed in [12]). Clb2-Cdk1 initially activates Swe1 through direct phosphorylation, thus leading to its own inhibition [6]. However, this phosphorylation primes further phosphorylations on Swe1 by multiple kinases, including Cdc5, that eventually target it to degradation [13].

Another key aspect of mitotic entry concerns the downregulation of phosphatases counteracting cyclinB/Cdk1 activity. In budding yeast, the Cdc14 phosphatase promotes inactivation of cyclinB/Cdk1 at the end of mitosis and reverses their phosphorylations [14], [15]. In interphase Cdc14 is kept inactive in the nucleolus [16], [17]. In all other eukaryotic cells analysed to date, phosphorylation events by mitotic CDKs are reversed mainly by PP2A and, to a lesser extent, by PP1 phosphatases (reviewed in [18]). Phosphatases are often protein complexes containing catalytic and regulatory subunits that confer substrate specificity. Core PP2A complexes are made of one catalytic (C subunit), one scaffold (A subunit) and one of many regulatory subunits (B subunit) [19]. Additional proteins, like the conserved Tap42, can also bind to PP2A and modulate its activity (reviewed in [20]). The B55 regulatory subunit, which exists in several isoforms, confers specificity of PP2A complexes towards Cdk1-dependent phosphorylation sites [21], suggesting that PP2A-B55 is the most relevant phosphatase in many eukaryotes for reversing Cdk1-dependent phosphorylations. Consistently, PP2A-B55δ prevents mitotic entry in *Xenopus* egg extracts [22], [23] and PP2A-B55α promotes mitotic exit in human cells [24].

In the past few years, the Greatwall protein kinase has emerged as a key factor for restraining PP2A-B55 activity in mitosis and allowing mitotic entry. In *Xenopus* egg extracts Greatwall is required for mitotic entry and to maintain the mitotic state [23], [25], [26], [27]. Depletion of Greatwall in *Drosophila* neuroblasts leads to mitotic defects that are compatible with a role of Greatwall in promoting entry into mitosis [28], [29]. Two closely related regulatory proteins, α-endosulfine (ENSA) and Arpp19, bind and inhibit PP2A-B55δ upon phosphorylation by Greatwall on a specific serine residue [30], [31]. Inactivation of *Drosophila* endosulfine leads to mitotic defects similar to those caused by Greatwall inactivation [32], while depletion of Arpp19 arrests *Xenopus* egg extracts in G2 [30], [31]. Indeed, Greatwall-dependent phosphorylation of α-endosulfine and Arpp19 promotes mitotic entry both by maintaining high levels of phosphorylated Cdk1 substrates and by feeding the Cdk1 autoregulatory loop (reviewed in [33], [34]).

In stark contrast to other organisms, budding yeast PP2A^Cdc55^ promotes, rather than prevents, timely entry into mitosis by participating in the positive feedback loop for Cdk1 activation (reviewed in [20]). Indeed, PP2A^Cdc55^ opposes the initial Swe1 phosphorylation by Cdk1, which stimulates Cdk1-Tyr19 inhibitory phosphorylation [35]. In addition, PP2A^Cdc55^ dephosphorylates and activates Mih1 [36]. Consistently, mutants defective in PP2A^Cdc55^ activity accumulate high levels of Tyr19-phosphorylated Cdk1 [35], [37], [38], [39].

Budding yeast possesses two redundant endosulfines, called Igo1 and Igo2 (Initiation of G zero), that are phosphorylated by the Greatwall-related kinase Rim15 on the same serine (Ser64 of Igo1 and Ser63 of Igo2) that in *Xenopus* Arpp19 and ENSA is phosphorylated by Greatwall [40]. Deletion of *IGO1* and *IGO2*, as well as of *RIM15*, does not affect cell viability but compromises the establishment of the quiescence (G0) state. Indeed, Igo proteins phosphorylated by Rim15 bind to an mRNA decapping factor to shelter degradation of specific mRNAs during initiation of the quiescence program [40]. Furthermore, recent data indicate that Igo proteins help establishing the quiescence-specific transcriptional program through binding and inhibition of PP2A bound to its B subunit Cdc55 [41]. Similar to yeast, depletion of α-endosulfine by RNAi or deletion of its aminoacid sequence targeted by Greatwall has no obvious consequence on cell division and fertility in the worm *C. elegans* [42].

In this manuscript we report the roles of Rim15 and Igo proteins in the control of mitosis. In agreement with recently published data [41], we find that upon phosphorylation by Rim15 Igo1 binds to yeast PP2A^Cdc55^ and can inhibit its activity *in vitro*. Consistently, phosphorylated Igo1 promotes mitotic entry in *Xenopus* egg extracts similarly to ENSA and Arpp19, suggesting that endosulfines from different species are interchangeable. However, phenotypic analyses of yeast mutants lacking Rim15 or Igo proteins, together with biochemical data, indicate that yeast endosulfines behave *in vivo* as activators, rather than inhibitors, of PP2A^Cdc55^ and contribute to timely activation of mitotic CDKs. Our data indicate that Igo proteins help establishing the positive feedback loop for Cdk1 activation and retaining PP2A^Cdc55^ in the cytoplasm [43], thereby promoting mitotic entry. We propose that endosulfines are accessory subunits of PP2A-B55 complexes that can contribute to their activation while inhibiting their phosphatase activity, to meet specific mitotic features in different organisms.

## Results

### Phosphorylation of Igo1 by Rim15 stimulates Igo1 interaction with PP2A^Cdc55^ in late S/G2 phase

**Figure 1:**
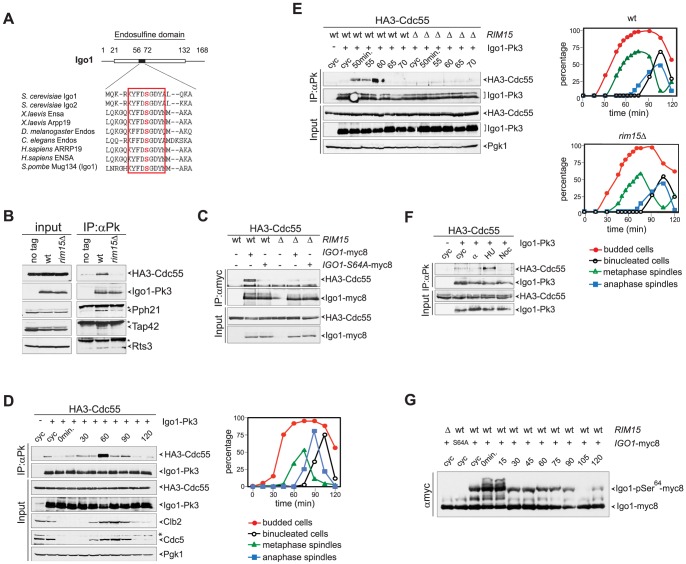
Igo1 interacts with PP2A^Cdc55^ upon Ser64 phosphorylation in lateS/G2 phase of the cell cycle. **A**. Primary sequence alignment of endosulfines from different species around the serine phosphorylated by Greatwall/Rim15. **B**. Cell extracts from wild type and *rim15*Δ cells expressing HA3-Cdc55 and Igo1-Pk3 as only sources of Cdc55 and Igo1 were subjected to immunoprecipitation with anti-Pk antibodies. The amount of HA3-Cdc55, Pph21, Tap42 and Rts3 co-immunoprecipitating with Igo1-Pk3 was assessed by western blot analysis. An extract from an Igo1-untagged strain was used as negative control (no tag). The asterisk marks the IgG in the IPs. **C**. Co-immunoprecipitations of HA3-Cdc55 with either wild type Igo1-myc8 or Igo1-S64A-myc8, in the presence or absence of *RIM15*. **D–E**. Cycling (cyc) cultures of cells expressing HA3-Cdc55 and Igo1-Pk3 was arrested in G1 by α-factor and released in fresh medium at 25°C. At different time points after release cell samples were collected to analyse the interaction between Igo1-Pk3 and HA3-Cdc55 after immunoprecipitation with anti-Pk antibodies (left, IP:αPk), the protein levels of HA3-Cdc55, Igo1-Pk3, Clb2, Cdc5 (left, Input), as well as the kinetics of budding, DNA replication (by FACS, data not shown), spindle formation/elongation and nuclear division (right graph). Pgk1 was used as loading control for the western blot. **F**. Cycling (cyc) cells were arrested in G1 by alpha factor or in S phase by hydroxyurea (HU) or in mitosis by nocodazole (Noc) to analyse the interaction between Igo1-Pk3 and HA3-Cdc55 after immunoprecipitation with anti-Pk antibodies. **G**. A cycling (cyc) culture of cells expressing Igo1-myc8 was arrested in G1 by alpha factor and released into the cell cycle at 25°C. At the indicated time points cell samples were collected for FACS analysis of DNA contents (not shown) and to prepare TCA protein extracts that were run on precast Phos-tag gels to visualize the phosphorylation of Igo1 by mobility shift. Extracts from *rim15Δ* and Igo1-S64A-myc8 cells were loaded as controls. Note that alpha factor induces an additional mobility shift likely corresponding to additional phosphorylations that are quickly lost upon cell cycle entry.

The budding yeast paralogous proteins Igo1 and Igo2 share significant homology with vertebrate endosulfines [44], especially around the serine that in endosulfines is phosphorylated by Greatwall (Gwl) (Fig. 1A). Indeed, Ser64 of Igo1 was shown to be phosphorylated by the yeast Greatwall-like kinase Rim15 [40]. To investigate if yeast endosulfines interact physically with PP2A in yeast cells, endogenous Igo1 was tagged at the C-terminus with 3 Pk epitopes and immunoprecipitated from yeast cells extracts. We then assessed the presence of various PP2A subunits in the immunoprecipitates. As shown in Fig. 1B, we found the catalytic subunit Pph21 and the Cdc55 regulatory subunit of PP2A to co-immunoprecipitate with Igo1-Pk3. In addition, Rts3 and Tap42, which associate with different PP2A phosphatases [45], [46], were also found in the immunoprecipitates. In contrast, we could not detect Rts1, the other major PP2A regulatory subunit, bound to Igo1-Pk3 (Fig. S1A). Based on several independent experiments, we estimated that about 1% of total Cdc55 is bound to Igo1 in asynchronous cycling cells. Interestingly, Igo1 association with PP2A subunits was dramatically impaired upon deletion of *RIM15*, suggesting that it is enhanced by Igo1 phosphorylation (Fig. 1B). Consistently, the interaction between HA-tagged Cdc55 and the mutant protein Igo1-S64A tagged with myc epitopes was markedly reduced compared to its wild type counterpart (Fig. 1C). Thus, efficient interaction between Igo1 and PP2A requires Rim15-dependent phosphorylation of Igo1 on Ser64. The residual binding between Igo1-S64A and Cdc55 appeared to be further impaired by *RIM15* deletion (Fig. 1C), suggesting that Rim15 might phosphorylate additional Igo1 residues besides S64 to stabilize the Igo1-PP2A^Cdc55^ complex. Indeed, recombinant Igo1-S64A could be still phosphorylated *in vitro* by human Greatwall, albeit inefficiently (Fig. S1B).

We next asked if Igo1 interaction with PP2A^Cdc55^ is regulated during the cell cycle. To this end, wild type cells co-expressing Pk-tagged Igo1 and HA-tagged Cdc55 were arrested in G1 by alpha-factor and released into fresh medium at 25°C. At different time points after release we analysed Igo1-Cdc55 interaction by co-immunoprecipitation, as well as other cell cycle parameters like budding, formation and elongation of bipolar spindles. As shown in Fig. 1D, a basal level of Cdc55 binding to Igo1 was detectable during most cell cycle stages but sharply peaked at 60 minutes from the G1 arrest. Based on the kinetics of bipolar spindle formation (starting at 60 minutes after release) and the appearance of the mitotic cyclin Clb2 and the polo kinase Cdc5 (peaking at 75 minutes after release), we conclude that Igo1-Cdc55 interaction is maximal in late S/G2 phase. The rise and fall in Igo1-Cdc55 interaction during the cell cycle was very sharp, as shown by a tighter time course, and depended on Rim15 (Fig. 1E). In addition, it was present at relatively high levels in cells treated with the replication inhibitor hydroxyurea, but not in cells arrested in G1 by alpha factor or arrested in mitosis upon spindle depolymerization by nocodazole (Fig. 1F). In spite of its cell cycle-regulated interaction with Cdc55, phosphorylation of Igo1 on Ser64 appeared to be constant throughout the cell cycle, as shown by Phos-tag phosphate affinity gel electrophoresis (Fig. 1G). Thus, Rim15-dependent phosphorylation of Ser64 is necessary for Igo1 binding to Cdc55, but other factors must be involved for this interaction to be maximal in late S or G2 phase.

### Yeast endosulfines can be phosphorylated by Gwl and induce mitotic entry in *Xenopus* egg extracts

**Figure 2:**
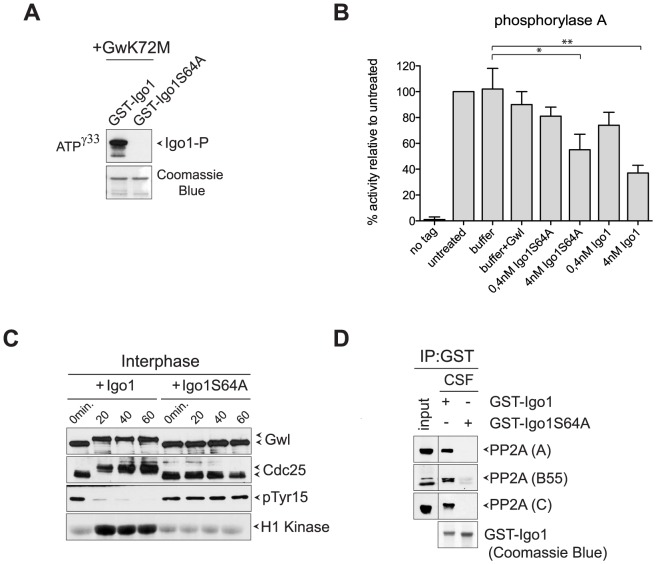
Yeast Igo1 induces mitotic entry in *Xenopus* egg extracts. **A**. Human hyperactive Gwl (Gwl-K72M) was used to phosphorylate in *vitro* GST-Igo1 and GST-Igo1S64A purified from bacterial cells. **B**. Bacterially purified GST-Igo1 or GST-Igo1S64A were phosphorylated *in vitro* by human Gwl and added at the indicated concentrations to PP2A^Cdc55^ complexes affinity-purified from yeast cells expressing HA-tagged Cdc55. The “no tag” control corresponds to the activity of an anti-HA affinity purification from cells expressing untagged Cdc55. **C**. Igo1 or Igo1S64A previously phosphorylated *in vitro* by Gwl were added to *Xenopus* egg interphase extracts at time 0. At the indicated time points aliquots of extracts were frozen in liquid nitrogen and subsequently analysed by western blot for Gwl and Cdc25 phosphorylation (causing a mobility shift in mitosis) and Cdk1 phosphorylation on Tyr15. Cdk1 kinase activity was measured using histone H1 as substrate. **D**. GST-Igo1 and GST-Igo1S64A were pulled down to assess their association with A, B55 and C subunit of PP2A by western analysis.

To test if yeast endosulfines inhibit PP2A catalytic activity like in other organisms, we affinity-purified PP2A^Cdc55^ complexes from yeast cells expressing HA-tagged Cdc55 and measured its associated phosphatase activity on the P-Ser/P-Thr substrate phosphorylase a [47]. Recombinant GST-tagged Igo1, but not its S64A mutant variant, could be readily phosphorylated by a hyperactive version of the Gwl kinase (Gwl-K72M) purified from baculovirus-infected insect cells (Fig. 2A), suggesting that Gwl and Rim15 are interchangeable for Igo1 phosphorylation *in vitro*. Consistent with previous results [41], addition of phosphorylated Igo1 to PP2A^Cdc55^ complexes inhibited their activity *in vitro* in a dose-dependent manner (Fig. 2B). The S64A mutation reduced, but did not abolish, the ability of Igo1 to inhibit PP2A activity (Fig. 2B).

To establish if Igo1 and Igo2 are functional orthologs of endosulfines, we asked if they could promote mitotic entry in *Xenopus* egg extracts. Addition of recombinant Igo1 phosphorylated *in vitro* by Gwl to *Xenopus* interphase extracts induced mitotic entry, as assessed by the appearance of phosphorylated forms of Gwl and Cdc25, the disappearance of Cdk1 inhibitory phosphorylation and raise in histone H1 kinase activity. In stark contrast, addition of Igo1-S64A had no effect (Fig. 2C). Since endosulfine-mediated mitotic entry under these conditions depends on inhibition of PP2A/B55, we asked if Igo1 could bind to PP2A in *Xenopus* egg extracts. Strikingly, wild type Igo1, but not Igo1-S64A, pulled down the catalytic (C), structural (A) and regulatory (B55) subunit of PP2A from CSF-induced mitotic extracts (Fig. 2D).

Thus, yeast endosulfines can inhibit the phosphatase activity of PP2A complexes and fulfill the role of their vertebrate counterparts in promoting mitotic entry.

### Lack of Rim15 or Igo proteins delays mitotic entry under temperature stress conditions by maintaining the inhibitory phosphorylation of Cdk1

To further investigate the possible role of Rim15 and Igo1,2 in cell cycle progression, we analysed the latter upon deletion of *RIM15* or *IGO1* and *IGO2*. Deletion of *RIM15* or *IGO1* and *IGO2* had no significant effect on the kinetics of cell division at physiological temperatures (25°C and 30°C, data not shown). However, *rim15*Δ and *igo1*Δ *igo2*Δ mutant cells where sensitive to thermal stress (14°C, 16°C and 38°C) and were hypersensitive to cell wall stressors, such as caffeine, calcofluor white and SDS (Fig. S2A).

**Figure 3:**
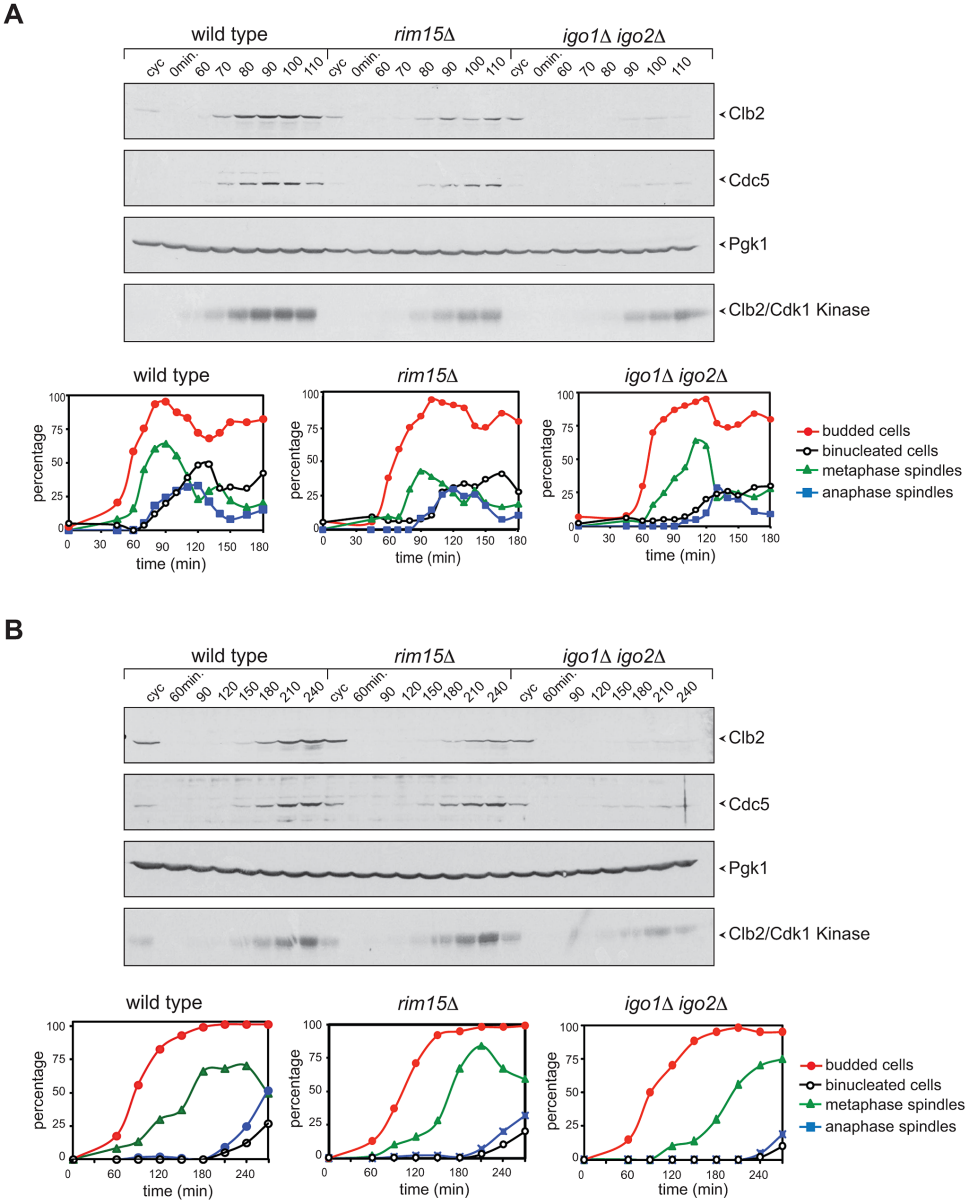
Deletion of *RIM15* or *IGO1* and *IGO2* delays mitotic entry under temperature stress. Cycling (cyc) cultures of wild type, *rim15Δ* and *igo1Δ igo2Δ* cells were arrested in G1 by α-factor and released in fresh medium at 38°C (**A**) or 16°C (**B**). At the indicated time points cells were collected for FACS analysis of DNA contents (not shown), kinetics of budding, spindle assembly/elongation and nuclear division (graphs), as well as to prepare protein extracts for western blot analysis of Clb2 and Cdc5 levels and for Clb2/Cdk1 kinase activity using histone H1 as substrate. Pgk1 was used as loading control.

We compared the cell cycle progression of *rim15*Δ and *igo1*Δ *igo2*Δ mutants to that of wild type cells at high and low temperatures. Cells were grown at 25°C, arrested in G1 by alpha-factor and then released in the cell cycle at 38°C. Although the kinetics of budding were similar in the three strains, mitotic events, such as spindle formation, spindle elongation and nuclear division, were delayed by 10–30 minutes in *rim15*Δ and *igo1*Δ *igo2*Δ cells relative to wild type. In addition, accumulation of the polo kinase Cdc5, as well as of the mitotic cyclin Clb2 and its associated kinase, were affected by *RIM15* deletion and, even more pronouncedly, by *IGO1* and *IGO2* deletion (Fig. 3A). A similar experiment showed that deletion of *RIM15* and *IGO1* and *IGO2* delayed mitosis also at 16°C, as shown by analysis of the same cell cycle markers above (Fig. 3B). Also at low temperature the mitotic defects of *igo1*Δ *igo2*Δ cells were more pronounced than those of *rim15*Δ cells. Taken together, these data indicate that Rim15 and its targets Igo1 and Igo2 are required for timely mitotic entry and mitotic progression under temperature stress, with Igo proteins having a more prominent role than Rim15 in this process. Consistent with a conserved function for Igo proteins and endosulfines in promoting mitotic entry, at least under certain conditions, expression of human Arpp19 or human ENSA from the *IGO1* promoter partially rescued the temperature-sensitivity of *igo1*Δ *igo2*Δ cells at 37°C (Fig. S2B).

### Rim15 and Igo1,2 promote mitotic entry by impinging on the positive feedback loop of Cdk1 activation

**Figure 4:**
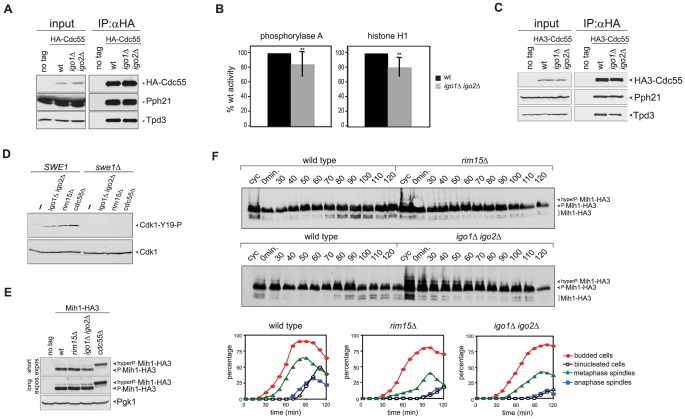
Igo proteins are required for full PP2A^Cdc55^ activity and timely Cdk1-Tyr19 and Mih1 dephosphorylation. **A–B**. HA-Cdc55 expressed from the *TPI1* promoter was immunoprecipitated from lysates of wild type (wt) or *igo1Δ igo2Δ* cells. Immunoprecipitates were analysed by western blot for the presence of HA-Cdc55, Pph21 and Tpd3 (A) and in parallel assayed for phosphatase activity using phosphorylase a (n = 12) and histone H1 (n = 5) as substrates (B). Statistical significance of differences was assesses by Student's *t*-test (** *p*<0.01). **C**. HA-Cdc55 expressed at endogenous levels from the *CDC55* promoter was immunoprecipitated from lysates of wild type (wt) or *igo1Δ igo2Δ* cells. Immunoprecipitates were analysed by western blot for the presence of HA-Cdc55, Pph21 and Tpd3. **D**. Cells with the indicated genotypes were arrested in G1 by α-factor and released for 2 hours in the presence of nocodazole. The levels of Tyr19-phosphorylated Cdk1 (Cdk1-Y19-P) were measured by western blot analysis. **E**. Total lysates of wild type, *rim15Δ*, *igo1Δ igo2Δ* and *cdc55Δ* cells expressing HA-tagged Mih1 (Mih1-HA3) and logarithmically growing at 25°C were analysed by western blot with anti-HA antibodies. **F**. Logarithmically growing (cyc) cultures of the same strains as in (A) were arrested in G1 by alpha factor and released in the cell cycle at 38°C to analyse at the indicated time points the electrophoretic mobility of Mih1-HA3 by western blot. Cell samples were also collected at the indicated time points to measure DNA contents by FACS analysis (not shown), as well as kinetics of budding, spindle assembly/elongation and nuclear division (graphs).

Since yeast PP2A^Cdc55^ promotes timely entry into mitosis, the genetic data above raised the possibility that *in vivo* Igo proteins contribute to PP2A^Cdc55^ activation. We therefore set out to measure PP2A^Cdc55^ catalytic activity in wild type and *igo1*Δ *igo2*Δ cells. To this end, we immunoprecipitated HA-Cdc55 and tested the activity of the associated phosphatase using phosphorylase a and histone H1 as substrates. As shown in Fig. 4A, anti-HA immunoprecipitates contained the Pph21 catalytic and the Tpd3 scaffold subunits of PP2A. Remarkably, lack of Igo1 and Igo2 caused a small (15–20%) but significant decrease on PP2A^Cdc55^ activity on both phosphorylase a and histone H1 (Fig. 4B). No further decrease in PP2A^Cdc55^ activity was observed by incubating *igo1*Δ *igo2*Δ cells at the restrictive temperature (38°C, data not shown). Thus, although Igo proteins are PP2A^Cdc55^ inhibitors, *in vivo* they sustain full PP2A^Cdc55^ activity. However, they did not seem to affect the basic composition of the hetero-trimeric PP2A^Cdc55^ complex, as shown by the similar levels of interaction between endogenous HA-tagged Cdc55 and the PPh21 and Tpd3 subunits in wild type and *igo1*Δ *igo2*Δ cells (Fig. 4C).

Deletion of *CDC55* causes an accumulation of Tyr19-phoshorylated Cdk1 and delays mitotic entry [37], [39]. Our finding that Igo1 and Igo2 are required for full PP2A^Cdc55^ activity and for timely mitotic entry under temperature stress conditions prompted us to test if Rim15 and Igo proteins regulate Cdk1 phosphorylation on Tyr19. To test if the levels of Tyr19 phosphorylation on Cdk1 were misregulated in the absence of Rim15 or Igo proteins, wild type, *igo1*Δ *igo2*Δ and *rim15Δ* mutant cells, as well as *cdc55*Δ cells used as control, were arrested in G1 and released in the presence of the microtubule-depolymerizer nocodazole at 25°C, i.e. at a temperature where *RIM15* and *IGO1/2* are not required for mitotic entry (data not shown). The phosphorylation status of Cdk1 was monitored by western analysis using a phospho-specific antibody that recognizes phosphorylated Cdk1-Y19. In agreement with previous reports [36], [39], [43], phosphorylation of Cdk1-Y19 was barely detectable in wild-type cells and high in *cdc55*Δ mutant cells under these conditions (Fig. 4D). Similarly, Cdk1-Y19 phosphorylation was increased in *igo1*Δ *igo2*Δor *rim15Δ* mutant cells relative to wild type cells and was fully abolished by *SWE1* deletion (Fig. 4D). Similar results were obtained under conditions of thermal stress (data not shown). Thus, Rim15 and Igo proteins are required for timely Cdk1 dephosphorylation, even in conditions where they do not appear to regulate mitosis.

We therefore analysed the levels and phosphorylation state of Swe1 during the cell cycle. Swe1 is phosphorylated in early mitosis by Clb2-Cdk1, which stimulates Swe1's ability to bind, phosphorylate and inhibit mitotic CDKs [6]. This initial Swe1 phosphorylation is opposed by PP2A^Cdc55^ [35]. Later on during mitosis, Swe1 gets hyperphosphorylated and eventually degraded [13], thus feeding the positive feedback loop. We assayed Swe1 levels and phosphorylation in synchronized *rim15Δ* and *igo1*Δ *igo2*Δ cells released from G1 in the presence of nocodazole at 25°C. As shown in Fig. S3A, HA-tagged Swe1 (Swe1-HA3) accumulated at intermediate phosphorylation levels and got more slowly hyperphosphorylated in the absence than in the presence of Rim15 (Fig. S3A). Similar data were obtained in cells lacking Igo1 and Igo2 (data not shown). Delayed appearance of Swe1 hyperphosphorylated forms did not seem to be further affected by deletion of *RIM15* or *IGO1* and *IGO2* upon release of G1 cells at the restrictive temperature of 38°C (Fig. S3B). Thus, Rim15 and Igo proteins contribute, like PP2A^Cdc55^, to timely Swe1 phosphorylation even in conditions where these proteins are apparently not required for timely mitotic entry.

Cdk1-Y19 phosphorylation is reversed by the Mih1 phosphatase, which in turn undergoes a PP2A^Cdc55^-dependent dephosphorylation that can be visualized by an increase in its electrophoretic mobility and correlates with mitotic entry [36]. We therefore asked if the levels of Mih1 phosphorylation are affected by deletion of *RIM15* or *IGO1* and *IGO2*. To this purpose, we expressed HA-tagged Mih1 (Mih1-HA3) in wild type, *rim15Δ* and *igo1*Δ *igo2*Δ mutant cells, as well as *cdc55*Δ cells used as control, and analysed Mih1 phosphorylation by western blot. Consistent with a previous report [36], most Mih1 was present in the cells in phosphorylated forms (Fig. 4E–F). Deletion of *RIM15* or *IGO1* and *IGO2* led to accumulation of hyperphosphorylated forms already at 25°C, albeit not to the same levels as deletion of *CDC55* (Fig. 4E). To analyse the transient appearance of the dephosphorylated, and presumably active, form of Mih1 during the cell cycle, wild type, *rim15Δ* and *igo1*Δ *igo2*Δ cells were arrested in G1 by alpha factor and released in the cell cycle at 38°C. Whereas dephosphorylated Mih1-HA3 started accumulating in wild type cells at 70–80 minutes after the release, coincident with the time of mitotic entry, its appearance was delayed in the absence of Rim15 or Igo proteins (Fig. 4F), in agreement with reduced PP2A^Cdc55^ activity. Thus, the phosphorylation state of both Swe1 and Mih1 is affected by deletion of *RIM15* or *IGO1/2*.

**Figure 5:**
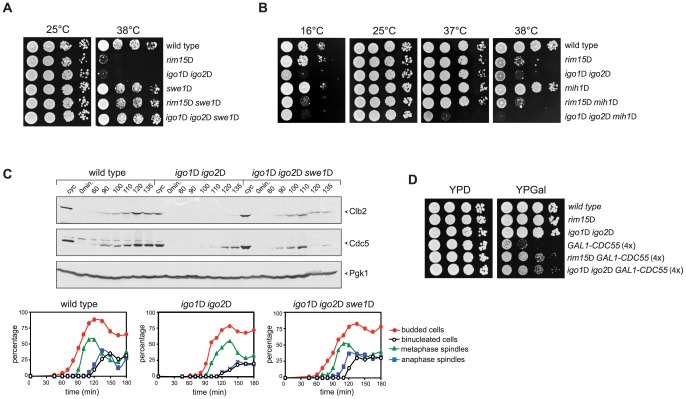
Deletion of *RIM15* or *IGO1* and *IGO2* affects the positive feedback loop for Cdk1 activation. **A–B**. Serial dilutions of strains with the indicated genotypes were spotted on YEPD plates and incubated for 48 hours at the indicated temperatures. **C**. Cycling (cyc) cultures of wild type, *igo1Δ igo2Δ* and *igo1Δ igo2Δ swe1Δ* cells were arrested in G1 by α-factor and released in fresh medium at 38°C. At the indicated time points cells were collected for FACS analysis of DNA contents (not shown), kinetics of budding, spindle assembly/elongation and nuclear division (graphs), as well as to make protein extracts for western blot analysis of Clb2 and Cdc5 levels. Pgk1 was used as loading control. **D**. Serial dilutions of strains with the indicated genotypes were spotted on YPD and YPGal plates and incubated at 25°C for 2 days.

Consistent with a role of Rim15 and Igo proteins in the regulation of Cdk1 phosphorylation, genetic analyses showed that lack of Swe1 fully rescued the temperature-sensitive growth defect of *igo1*Δ *igo2*Δ and *rim15*Δ mutant cells at 38°C (Fig. 5A). Expression of non-phosphorylatable Cdc28-Y19F also rescued the cold- and temperature-sensitivity of *igo1*Δ *igo2*Δ and *rim15*Δ mutants, although somewhat less efficiently than *SWE1* deletion (Fig. S4). Finally, *MIH1* deletion enhanced the temperature-sensitivity of *igo1Δ igo2Δ* cells at 37°C and caused a cold-sensitive growth phenotype to *rim15Δ* cells at 16°C (it should be noted that *igo1Δ igo2Δ* cells are sensitive to this temperature ontheirown,Fig. 5B).

We then asked if *SWE1* deletion could rescue the mitotic entry delay of *igo1*Δ *igo2*Δ under temperature stress. To address this question, we arrested wild-type, *igo1*Δ *igo2*Δ and *igo1*Δ *igo2*Δ*swe1*Δ cells in G1 and released them into fresh medium at 38°C. Under these conditions, *igo1*Δ *igo2*Δ cells showed a marked delay in the accumulation of Clb2 and Cdc5 and in spindle elongation relative to wild type cells (Fig. 3A and 5C). Strikingly, *SWE1* deletion suppressed these mitotic defects and restored the normal kinetics of Clb2 and Cdc5 accumulation during the cell cycle (Fig. 5C). Altogether, these data suggest that Rim15 and Igo proteins regulate the phosphorylation state of Cdk1 for mitotic entry, presumably by affecting the PP2A^Cdc55^–dependent regulation of Swe1 and Mih1, and indicate that excessive Cdk1 inhibitory phosphorylation is responsible for the mitotic delay of *igo1*Δ *igo2*Δ cells.

If budding yeast endosulfines were required for full PP2A^Cdc55^ phosphatase activity, deletion of *RIM15* or *IGO1* and *IGO2* should rescue the toxic effects caused by *CDC55* overexpression, which prevents mitotic progression [48]. Consistent with our previous findings, cells carrying four integrated copies of a galactose-inducible *GAL1-CDC55* construct were mostly unable to divide on galactose-containing plates (YPGal, Fig. 5D). Loss of Rim15 or Igo1/2 efficiently suppressed this lethality (Fig. 5D) without affecting the levels of *CDC55* overexpression (data not shown).

Altogether, these data indicate that Rim15 and Igo proteins contribute to activation of PP2A^Cdc55^, at least for what concerns some of its mitotic targets, thereby tuning the levels of Cdk1 phosphorylation to promote mitotic entry.

### Igo proteins influence Cdc55 subcellular localization

**Figure 6:**
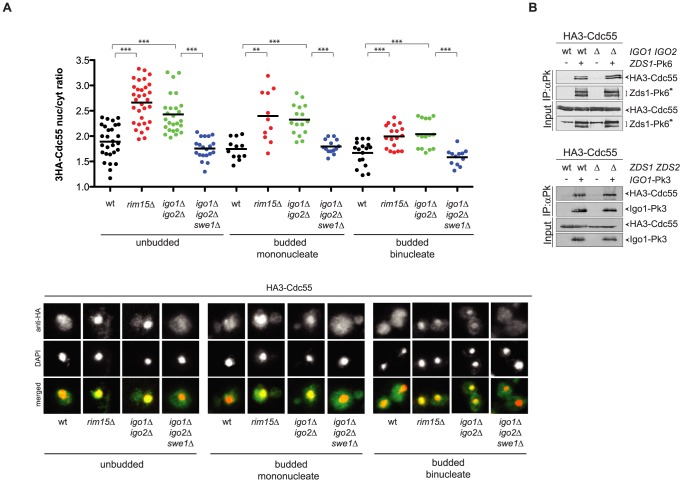
Igo1 and Igo2 control the subcellular localization of PP2A^Cdc55^ independently of Zds proteins. **A**. Localization of HA3-Cdc55 was analysed by indirect immunofluorescence with anti-HA antibodies after formaldehyde fixation of cycling cultures of wild type, *rim15Δ igo1Δ igo2Δ*and *igo1Δ igo2Δ swe1Δ*cells. Cells were scored according to their cell cycle stage: unbudded (G1), budded mononucleated (S, G2, early M) and budded binucleated (late M). At least 15 cells were scored for each class. **B**. Interaction between Zds1-Pk6 and HA3-Cdc55 in the presence or absence of Igo proteins (left panel) and interaction between Igo1-Pk3 and HA3-Cdc55 in the presence or absence of Zds proteins (right panel) was assessed by immunoprecipitation with anti-Pk antibodies followed by western blot analysis with anti-HA and anti-Pk antibodies. The asterisk marks a protein aspecifically recognized by the anti-Pk antibodies in the total extracts (input) and that co-migrates with Zds1-Pk6.

The apparently opposite impact of Igo proteins on PP2A regulation *in vitro* and *in vivo* (i.e. inhibition versus activation, respectively), raised the possibility that in yeast Igo1/2 modulate PP2A activity at a different/additional level. For instance, the Zds1 and Zds2 proteins, which bind PP2A^Cdc55^ in a stoichiometric complex and inhibit its activity *in vitro* [49], [50], were recently shown to regulate the subcellular localization of Cdc55. More specifically, Zds1/2 induce the nuclear export of Cdc55 into the cytoplasm, which in turn promotes mitotic entry [43]. We therefore asked if Rim15 and Igo proteins might play a similar role in controlling Cdc55 localization. HA-tagged Cdc55 was detected by indirect immunofluorescence in wild type and *igo1*Δ *igo2*Δ cells at various cell cycle stages, namely in G1 (unbudded cells), in S, G2 and early M phases (budded mononucleated cells) and in middle/late M phase (budded binucleated cells). As previously shown [43], Cdc55 was localized in both the nucleus and the cytoplasm. However, it was significantly more concentrated in the nucleus of *rim15*Δand *igo1*Δ *igo2*Δ cells than in the wild type in all cell cycle stages (Fig. 6A). Strikingly, deletion of *SWE1* restored the normal nuclear/cytoplasmic ratio of Cdc55 (Fig. 6A), strongly indicating that the altered subcellular distribution of Cdc55 in mutants lacking Rim15 or Igo proteins is a consequence, rather than a cause, of misregulated positive feedback loop for Cdk1 activation.

The similar phenotype of *igo1*Δ *igo2*Δ and *zds1*Δ *zds2*Δ cells with respect to Cdc55 localization raised the possibility that Igo and Zds proteins work in concert and/or are part of the same complex. We therefore decided to analyse the possible interdependence between Igo and Zds proteins for their interaction with Cdc55. As previously shown, Pk-tagged Zds1 efficiently co-immunoprecipitated HA-tagged Cdc55 from extracts obtained from cycling cells. Interaction between Zds1 and Cdc55 was not affected by deletion of *IGO1* and *IGO2* (Fig. 6B). Similarly, co-immunoprecipitation of HA-tagged Cdc55 with Pk-tagged Igo1 was unaffected by deletion of *ZDS1* and *ZDS2*. It is worth noting that in *zds1*Δ *zds2*Δ cells the mobility shift of Cdc55, which was previously shown to be due to phosphorylation [47], disappears, indicating another possible way for Zds proteins to regulate PP2A^Cdc55^ activity besides its subcellular localization. Altogether, these data suggest that Igo and Zds proteins bind independently to PP2A^Cdc55^ and might regulate its activity in an independent manner. Consistent with this conclusion, deletion of *IGO1* and *IGO2* caused synthetic sickness at high temperatures when combined with deletion of *ZDS1* and *ZDS2* (Fig. S5).

## Discussion

### Conservation of the Greatwall-Endosulfine-PP2A regulatory module

In several organisms endosulfine-like proteins bind to PP2A-B55 upon Greatwall-dependent phosphorylation of a conserved serine and inhibit it [33], [34]. Recent data showed that in quiescent yeast cells Rim15 phosphorylates the paralogous endosulfines Igo1 and Igo2, which in turn promote the transcription of specific nutrient-regulated genes by direct inhibition of the phosphatase PP2A^Cdc55^ [41]. We show here that Igo1 phosphorylated on Ser64 by Rim15 interacts also during the unperturbed cell cycle with the catalytic subunit of PP2A (Pph21), the B regulatory subunit Cdc55, Tap42 and Rts3 and that phosphorylated Igo1 can bind PP2A complexes in yeast and *Xenopus* egg extracts. PP2A has multiple functions during the cell cycle (reviewed in [20]). In yeast it associates with two major and alternative B subunits, Cdc55 and Rts1. Rts1 does not appear to interact with Igo1 in our co-immunoprecipitations. Cdc55 has been involved in many cellular processes, such as mitotic entry and exit, morphogenesis and cytokinesis, mitotic checkpoints and stress response [35], [36], [38], [39], [48], [51], [52], [53], [54], [55], [56]. Of particular interest is the presence in our Igo1 immunoprecipitates of the essential Tap42 subunit, the orthologue of human PP2A-associated α4/IgBP1. Tap42 associates to the catalytic subunit of PP2A and PP2A-like phosphatases independently of the A and B subunit during logarithmic growth as opposed to stationary phase, suggesting that its interaction with PP2A phosphatases is regulated by nutrients [57]. Consistently, Tap42 phosphorylation, which mediates its interaction with PP2As, depends on the phosphatidylinositol-related kinases Tor1 and Tor2 (Target of rapamycin), which regulate cell growth in response to nutrient availability and cell stress [58]. The last PP2A subunit that we found in Igo1 immunoprecipitates is Rts3, a poorly characterized protein that was found to interact with different PP2A and PP2A-like complexes [45], [59], [60] and whose deletion causes sensitivity to caffeine [61], which in turn inhibits the Tor complex TORC1 [62].

The peak of interaction between Igo1 and Cdc55 is cell cycle-regulated and peaks in late S or G2 phase (note that it is not possible to discriminate between late S and G2 phase in budding yeast due to the lack of specific markers), i.e. when PP2A^Cdc55^ participates to the positive feedback loop for Cdk1 activation (see below). In agreement with recently published data [41], we show that mutation of Igo1 Ser64, which is targeted by Rim15 [40], and deletion of *RIM15* reduce significantly Igo1 interaction with PP2A^Cdc55^, indicating that, like in higher organisms, endosulfine phosphorylation is required for its interaction with PP2A. In spite of its cell cycle-regulated interaction with PP2A^Cdc55^, Igo1 phosphorylation on Ser64 appears to be constitutive during the cell cycle, raising the interesting possibility that cell cycle-controlled factors are involved to make the Igo1-Cdc55 binding periodic. Along the same line, it is also interesting to notice that phosphorylation of Ser64 of Igo1 and Ser63 of Igo2 is stimulated after inhibition of Cdk1 [63], suggesting that Cdk1 activation in early mitosis may cause the dissociation of Igo-PP2A^Cdc55^ complexes.

The evolutionary conservation of the Greatwall-Endosulfine pathway is further strengthened by the finding that yeast Rim15 can phosphorylate *in vitro* human ENSA and Arpp19 [40], whereas human and *Xenopus* Greatwall can phosphorylate yeast Igo1 (this manuscript), indicating that Rim15 and Igo proteins are the true counterparts of vertebrate Greatwall and endosulfines, respectively. Thus, the Greatwall-Endosulfine-PP2A regulatory module appears to be conserved in all organisms analysed to date, with the notable exception of the nematode *C. elegans* where an obvious Greatwall-like kinase seems to be missing [42].

### Are endosulfines positive or negative regulators of PP2A?

ENSA and Arpp19 inhibit PP2A activity in *Xenopus* egg extracts [30], [31]. Inhibition of PP2A by endosulfines is in turn essential for mitotic entry in *Xenopus* and human cells and for mitotic progression in *Drosophila* [23], [27], [29], [30], [31], [64]. Similarly, we and others [41] find that phosphorylated Igo1 inhibit PP2A activity *in vitro* and induces interphase *Xenopus* egg extracts to enter mitosis, indicating that budding yeast endosulfines can inhibit PP2A, like their vertebrate counterparts. PP2A^Cdc55^ inhibition in rapamycin-treated yeast cells is important to establish a quiescence-specific transcriptional program [41]. We provide several lines of evidence indicating that Rim15 and Igo proteins also contribute to activate PP2A^Cdc55^ for mitotic entry *in vivo*. First, lack of Igo1 and Igo2 causes a slight reduction, rather than an increase, in PP2A^Cdc55^ phosphatase activity. Taking into account that only about 1% of Cdc55 is bound to Igo1 (and presumably a similar fraction of Cdc55 is bound to Igo2), the 15–20% reduction in PP2A^Cdc55^ activity in cells lacking Igo proteins would imply a prominent role for this proteins in the full activation of PP2A^Cdc55^ complexes. Second, deletion of *RIM15* or *IGO1* and *IGO2* delays accumulation of active cyclinB/Cdk1 and dephosphorylation of Cdk1 Tyr19, similarly to inactivation of PP2A^Cdc55^ [35], [39]. This is accompanied by a misregulation in the phosphorylation of the Swe1 kinase and the dephosphorylation of the Mih1 phosphatase during the cell cycle, which are both controlled by PP2A^Cdc55^ [35], [36]. Third, the toxic effects caused by *CDC55* overexpression [48] are rescued by *RIM15* or *IGO1* and *IGO2* deletion. Yet, not only phosphorylated Igo1 can inhibit PP2A activity *in vitro*, but expression of human ENSA or Arpp19 rescues the temperature-sensitivity of *igo1Δ igo2Δ* cells, suggesting that vertebrate and yeast endosulfines are interchangeable for their mitotic function(s). Therefore, the apparently opposite modes of PP2A regulation by endosulfines in yeast versus vertebrates are unlikely linked to intrinsic differences in the structure of endosulfines and/or in their ability to bind and inhibit PP2A. Several other examples of proteins that behave as activators or inhibitors of their binding partner(s) depending on the organism and/or the conditions have been reported. Similarly to Igo1 and Igo2, budding yeast Zds proteins were shown to inhibit PP2A^Cdc55^ phosphatase activity [49] and to promote efficient mitotic entry through nuclear export of Cdc55 and Cdc55-dependent regulation of the Cdk1 positive feedback loop [43], [50], [65]. Tap42 has been proposed to work as activator and inhibitor of PP2A [57], [66], [67], [68]. Finally, securin is both an inhibitor and a chaperone of separase for the regulation of sister chromatid splitting in anaphase. Depending on the organism, securin depletion/inactivation prevents sister chromatid separation or causes premature anaphase onset (reviewed in [69]). We speculate that Igo proteins might be chaperones for PP2A^Cdc55^ and assist its proper folding while keeping the complex inhibited. The presence of phosphorylated substrates could competitively bind to PP2A^Cdc55^ and overcome its inhibition. The exact biochemical mechanism of PP2A-B55 inhibition by endosulfines is currently unknown and will be likely clarified by structural and biochemical data.

### The function of Rim15 and Igo proteins in the control of mitosis

Consistent with a positive role of Igo proteins in PP2A^Cdc55^ regulation for mitosis, we find that the phosphatase activity of PP2A^Cdc55^ complexes in *igo1*Δ *igo2*Δ cells is reduced compared to wild type cells. This results in delayed phosphorylation of Swe1 and Mih1, which are known targets of PP2A^Cdc55^ [35], [36], thus preventing the sharp rise in CDK activity during mitotic entry. Indeed, *rim15Δ* and *igo1Δ igo2Δ* cells are unable to timely dephosphorylate Tyr19 of Cdk1 and to efficiently raise cyclinB/Cdk1 activity in mitosis, resulting in a delayed spindle elongation, which requires high cyclinB/Cdk1 levels [70]. The observation that *rim15Δ* and *igo1Δ igo2Δ* mutants display lower activity of PP2A^Cdc55^ complexes, nuclear retention of Cdc55 and accumulation of Tyr19-phosphorylated Cdk1 at physiological temperatures (e.g. 25°C), while progressing through mitosis with normal timing, is somewhat puzzling. Since mitotic defects of *rim15Δ* and *igo1Δ igo2Δ* mutants become apparent only under stress conditions, it is possible that the physiological state of cells can make them differentially responsive to increased levels of Tyr19-phosphorylated Cdk1. Deletion of *SWE1* or expression of the non-phosphorylatable Cdc28-Y19F mutant protein rescues the temperature-sensitive growth and mitotic defects of *rim15Δ* and *igo1Δ igo2Δ* cells, whereas deletion of *MIH1* aggravates them. The role of PP2A^Cdc55^ in the nucleolar retention and inhibition of Cdc14 [52], which is the major CDK-counteracting phosphatase in yeast [15], might also be affected by lack of endosulfines and contribute to timely mitotic entry.

**Figure 7:**
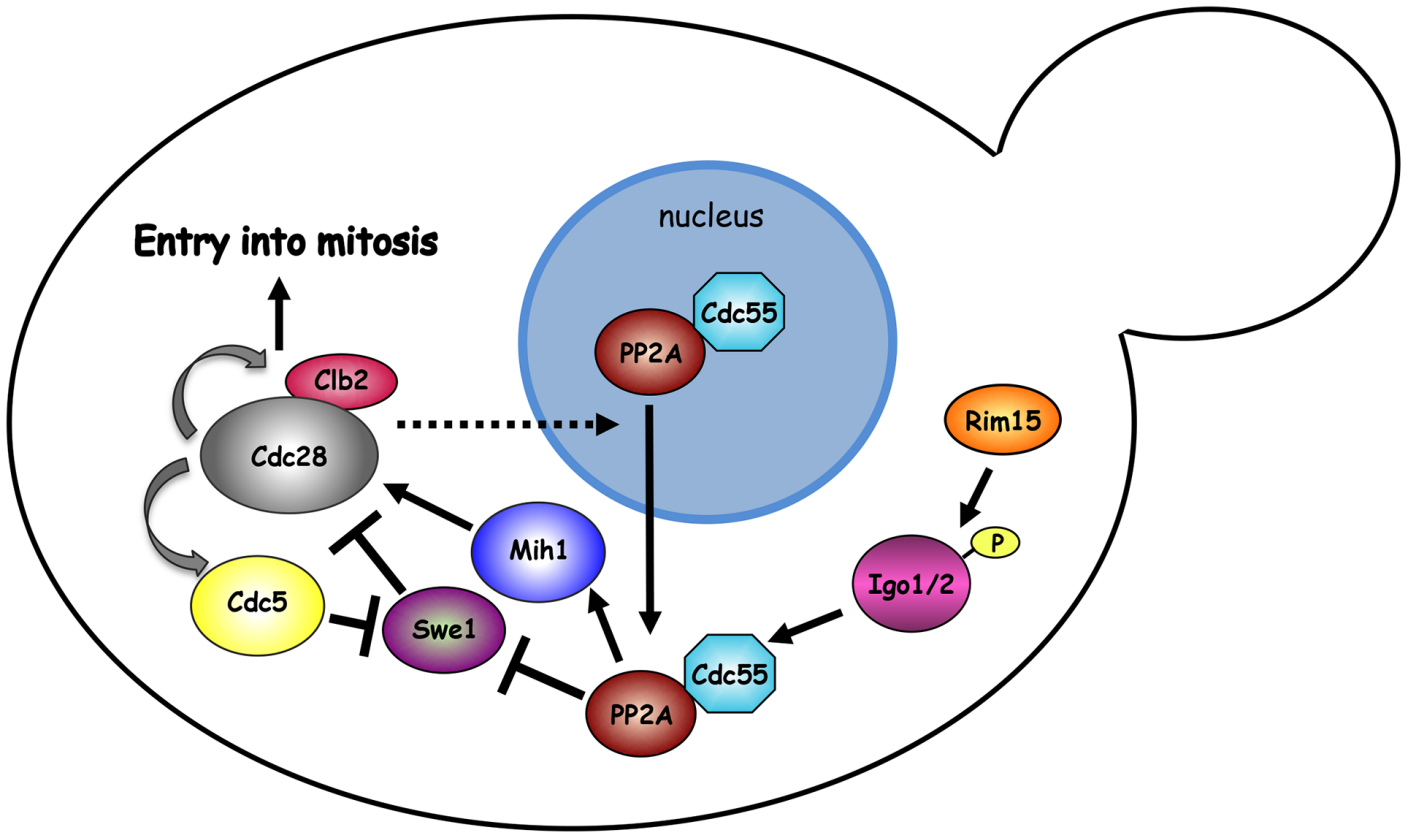
Model. The mitotic CDK Clb2-Cdk1 is part of a positive feedback loop where it contributes to its own activation by promoting a transcriptional mitotic program for its own expression and that of the polo kinase Cdc5. Although Swe1 phosphorylation by Clb2-Cdk1 promotes Tyr19 phosphorylation, and thereby inhibition, of Cdk1 (not depicted), it primes further Swe1 phosphorylations by several other kinases, including Cdc5, that finally target Swe1 to degradation. Initial Swe1 phosphorylation by Cdk1 is counteracted by the PP2A^Cdc55^ phosphatase, which also dephosphorylates and activates Mih1, thereby participating in the positive feedback loop for Cdk1 activation. The endosulfines Igo1 and Igo2, upon phosphorylation by Rim15, bind to PP2A^Cdc55^ and contribute to their activation and nuclear export. The observation that *SWE1* deletion restores normal localization of Cdc55 in *igo1Δ igo2Δ* mutant cells suggests that Swe1 and/or Clb2-Cdk1 might regulate the nuclear export of Cdc55 through an unknown mechanism. See text for further details.

The activity of PP2A^Cdc55^ has been recently proposed to be spatially regulated by nuclear export of Cdc55, which is regulated by Zds1 and Zds2 and promotes mitotic entry [43]. In this respect it is important to notice that a key determinant in Swe1 downregulation is its proteolysis, which requires Swe1 recruitment to the bud neck (i.e. out of the nucleus) [71], [72]. Thus, the cytoplasmic pool of PP2A^Cdc55^ might be instrumental to trigger efficient Swe1 degradation [39]. We show that the nuclear/cytoplasmic ratio of Cdc55 is increased throughout the cell cycle upon deletion of *RIM15* or *IGO1* and *IGO2*. The increased concentration of Cdc55 in the nuclei of *rim15*Δ and *igo1*Δ *igo2*Δ cells might be a consequence of decreased PP2A^Cdc55^ activity or be caused by a more direct function of yeast endosulfines in the control of Cdc55 nuclear-cytoplasmic shuttling. Our finding that *SWE1* deletion restores proper subcellular localization of Cdc55 in the absence of Rim15 and Igo proteins argues against the latter possibility. We thus favor the idea that yeast endosulfines only impact on PP2A^Cdc55^ activity, participating in the positive feedback loop for Cdk1 activation like their *Xenopus* counterparts [27]. CDK activity could in turn drive, directly or indirectly, Cdc55 export from the nucleus to the cytoplasm (Fig. 7). It is nevertheless possible that nuclear retention of Cdc55 in cells lacking Rim15 or endosulfines has a greater impact than the slight decrease in PP2A activity by itself on dephosphorylation of PP2A^Cdc55^ substrates, including Swe1 and Mih1. In fact, Cdc55 mislocalization could keep PP2A^Cdc55^ spatially segregated from its substrates. Further experiments will be required to understand the links between PP2A activity and its subcellular localization.

Similar to endosulfines, the Zds1 and Zds2 proteins bind tightly to PP2A^Cdc55^ [49], [50], affect its subcellular localization [43] and target PP2A^Cdc55^ activity to Mih1 [65], thus promoting timely mitotic entry [43], [50], [65]. In spite of the similarity, Igo and Zds proteins seem to work independently. Indeed, binding of Igo1 to Cdc55 does not require Zds1/2, whereas Zds1 interaction with Cdc55 does not require Igo proteins. In addition, deletion of *IGO1* and *IGO2* causes synthetic growth defects when combined to deletion of *ZDS1* and *ZDS2*.

In conclusion, our data emphasize the plasticity of the Greatwall-Endosulfine-PP2A module. This module is conserved, but it has adapted to account for the differences in mitotic regulation depending on the cellular context. In cells where PP2A antagonizes Cdk1 activity the Greatwall-endosulfine module only inhibits PP2A, whereas in budding yeast where PP2A promotes timely mitotic entry the same module also stimulates PP2A activity, resulting in both cases in a sharp mitotic switch [73].

## Materials and Methods

### Strains, media and reagents, genetic manipulations

All yeast strains (Table S1) were derivatives of W303 (*ade2-1, trp1-1, leu2-3,112, his3-11,15, ura3, ssd1*), except for strains used for phosphatase assays that were derivatives of S288C. Cells were grown in either synthetic minimal medium (SD) supplemented with the appropriate nutrients or YEP (1% yeast extract, 2% bactopeptone, 50 mg/l adenine) medium supplemented with 2% glucose (YEPD) or 2% galactose (YEPG). Unless differently stated, alpha factor was used at 4 µg/ml, hydroxyurea (HU) at 200 mM and nocodazole at 15 µg/ml. Standard techniques were used for genetic manipulations [74], [75]. Gene deletions were generated by one-step gene replacement [76]. One-step tagging techniques [77], [78] were used to tag at their C-terminus Igo1 (Igo1-Pk3), Swe1 (Swe1-HA3) and Mih1 (Mih1-HA3).

### Fluorescence microscopy

In situ immunofluorescence was performed on formaldehyde-fixed cells expressing HA-tagged Cdc55 using anti-HA monoclonal antibody (16B12, Covance Research Products), followed by indirect immunofluorescence using Cy3-conjugated goat anti-mouse antibody (GE Healthcare). To detect spindle formation and elongation, anti-tubulin immunostaining was performed with the YOL34 monoclonal antibody (Serotec) followed by indirect immunofluorescence using rhodamine-conjugated anti-rat antibody (Pierce Chemical Co). Digital images were taken with an oil 63X 1,4-0,6 HCX Plan-Apochromat objective (Zeiss) with a Coolsnap HQ2-1 charge-coupled device camera (Photometrics) mounted on a Zeiss AxioimagerZ1/Apotome fluorescence microscope controlled by the MetaMorph imaging system software. Fluorescence intensity of HA3-Cdc55 in the nucleus and the cytoplasm was quantified with ImageJ on a single focal plane. Significance of the differences between fluorescence intensities was statistically tested by means of a two-tailed *t*-test, assuming unequal variances.

### Protein extracts, immunoprecipitations and kinase assays

TCA protein extracts were prepared as previously described [79] for Phos-tag phosphate affinity gel electrophoresis (Wako, Fig. 1G) and to analyse the electrophoretic mobility of HA-tagged Swe1 and Mih1. For immunoprecipitations of Igo1-Pk3 or Zds1-Pk6, pellets from 50 ml yeast cultures (10^7^ cells/ml) were lysed at 4°C with acid-washed glass beads in lysis buffer (50 mM Tris-Cl pH 7.5, NaCl 150 mM, 10% glycerol, 1 mM EDTA, 1% NP40, supplemented with protein inhibitors (Complete, Roche), 1 mM Na-orthovanadate and 60 mM ß-glycero-phosphate). Total extracts were cleared by spinning at 12000 rpm for 10 minutes and quantified by NanoDrop. Same amounts of protein extracts were subjected to immunoprecipitation with an anti-Pk antibody (from AbD serotec) pre-adsorbed to protein A-sepharose. Inputs represent 1/50^th^ of the IPs final volumes.

For Clb2/Cdk1 kinase assays protein extracts were prepared as previously described [80]. 50 µg of extract were used for measuring kinase activity on histone H1 [81] and 30 µg for Western blot analysis.

Hyperactive human Gwl (Gwl-K72M) purified from baculovirus-infected cells or endogenous Gwl immunoprecipitated from CSF-treated *Xenopus* egg extracts [30] were used to phosphorylate in vitro GST-Igo1 purified from *E.coli* for the *in vitro* phosphorylation assay (Fig. 2A and Fig. S1B) and the mitotic entry experiment (Fig. 2C), respectively.

Yeast protein extracts were prepared according to [82] for western blot analysis. Proteins transferred to Protran membranes (Schleicher and Schuell) were probed with monoclonal anti-HA 12CA5, anti-Pgk1 (Molecular Probes), anti-Pk (AbD serotec), anti-Myc 9E10 or polyclonal Cdc5 antibodies (sc-6733 Santa Cruz), anti-Clb2 (sc-9071 Santa Cruz) or anti-phospho-cdc2 (pTyr15 Cell Signalling). Anti-Tap42, anti-Pph21 and anti-Rts3 antibodies were described [47]. Affinity-purified antibodies against Gwl and Cdc25 were also previously described [30]. Monoclonal anti-PP2A/C subunit (1D6) and anti-PP2A/A (6G3) antibodies were obtained from Upstate/Millipore and Cell Signalling, respectively. Polyclonal antibodies against PP2A/B55 were raised in rabbits and affinity-purified. Secondary antibodies were purchased from Amersham and proteins were detected by an enhanced chemiluminescence system according to the manufacturer.

### Protein phosphatase assay and treatment of PP2A-complexes with phosphorylated Igo1

For immunoprecipitation assays, yeast whole-cell extracts were prepared as described previously [83] except that lysis was performed using a Fastprep (MP Biomedicals, 1×40 s, 6 m/s). HA-tagged Cdc55 containing a flexible glycine linker (GL) after the HA epitope (GGGSGGGGS) was expressed from the strong constitutive *TPI1* promoter on an episomal centromeric plasmid [47]. HA-GL-Cdc55 was immunoprecipitated with anti-HA (clone 12CA5) antibodies cross-linked to BSA-coated protein A–Sepharose beads (GE Healthcare). Phosphatase activity of PP2A immunoprecipitates was assayed toward ^32^P-labeled phosphorylase a (Figs. 2B, 4A) or towards ^32^P-labeled histone H1 (Fig. 4B), as previously described [47], [83]. Immunoprecipitates were analyzed by 10% SDS-PAGE, immunoblotted and incubated with specific antibodies against Pph21 (rabbit pAB), Cdc55 (clone 9D3-H6) or Tpd3 (clone 5G2). The assay values (average of at least five independent experiments) are presented as a percentage of the wild-type strain activity, which was set at 100%. Values were normalized to the amount of Pph21 co-immunoprecipitated with HA-Cdc55 as determined by immunoblot and densitometer analysis using an Odyssey Infrared Imaging System (LI-COR, http://www.licor.com). For Figure 2B immunoprecipitates were split into 7 aliquots and in-vitro Greatwall-phosphorylated recombinant GST-Igo1 or GST-Igo1S64A (where kinase assays were carried out in 50 mM Tris pH7.2, 10 mM MgCl_2_, 1 mM ATP, 30°C for 30′) was added as indicated and phosphatase activity assays toward ^32^P-labeled phosphorylase a were conducted. The activities of the different samples are presented as percent of activity with respect to the activity of the untreated control, which was set to 100%. Data are presented as mean values ± standard deviation (SD), and were analyzed using two-tailed Student's *t*-test, assuming unequal variances. Differences with *p*-values lower than 0.05 were considered statistically significant (* *p*<0.05 ; ** *p*<0.01 ; *** *p*<0.001).

### Other techniques

Nuclear division was scored with a fluorescence microscope on cells stained with propidium iodide (Sigma Aldrich). Flow cytometric DNA quantification was performed according to [84] on a Becton-Dickinson FACSCalibur.

## Supporting Information

Figure S1 Igo1 interacts with the PP2A B subunit Cdc55 and not with its B′ subunit Rts1. A. Interaction between Igo1-Pk3 and either HA3-Cdc55 or Rts1-HA3 was assessed by immunoprecipitation with anti-Pk antibodies followed by western blot analysis with anti-HA and anti-Pk antibodies. B. Bacterially purified GST-Igo1 or GST-Igo1-S64A were phosphorylated *in vitro* by human Gwl using P^32^γATP. Phosphorylated Igo1 was visualized after SDS page and autoradiography.
(PDF)

Figure S2 Cells lacking Rim15 or Igo1 and Igo2 are sensitive to different stress conditions and their temperature-sensitivity is suppressed by expression of human endosulfines. A. Serial dilutions of strains with the indicated genotypes were spotted on YEPD plates and incubated at the indicated temperatures (top panel) or were spotted on YEPD containing 10 mM caffeine (Caff) or 0.01% calcofluor white (CFW) or 0.001% SDS and incubated at 25°C (bottom panel). B. Wild type and *igo1Δ igo2Δ* cells carrying the empty vector (YEp) or a high copy number plasmid carrying human *ARPP19* (YEp-*ARPP19*) or human *ENSA* (YEp-*ENSA*) under the control of the *IGO1* promoter [40] were spotted on SD-Ura plates and incubated for two days at 37°C.
(PDF)

Figure S3 Deletion of *RIM15* or *IGO1* and *IGO2* affects Swe1 hyperphosphorylation. Cycling (cyc) cultures of wild type, *rim15Δ* and *igo1Δ igo2Δ* cells expressing HA-tagged Swe1 (Swe1-HA3) were arrested in G1 by α-factor and released in fresh medium at 25°C in the presence of nocodazole (A) or at 38°C (B). At the indicated time points cells were collected for FACS analysis of DNA contents (not shown), kinetics of budding, spindle assembly/elongation and nuclear division (graphs), as well as to prepare TCA protein extracts for western blot analysis of Swe1-HA3 using anti-HA antibodies. Similar results were obtained in (A) for *rim15Δ* and *igo1Δ igo2Δ* cells (not shown).
(PDF)

Figure S4 Expression of the non-phosphorylatable Cdc28-Y19F mutant protein suppresses the temperature-sensitive growth defects of *rim15Δ* and *igo1Δ igo2Δ* cells. Serial dilutions of strains with the indicated genotypes were spotted on YEPD plates and incubated for 48 hours at the indicated temperatures.
(PDF)

Figure S5 Deletion of *IGO1* and *IGO2* causes synthetic sickness to cells lacking Zds proteins. A. A heterozygous *IGO1*/*igo1Δ IGO2/igo2Δ ZDS1*/*zds1Δ ZDS2/zds2Δ* diploid strain was induced to sporulate and its meiotic segregants were analysed in 103 independent tetrads for the presence of genetic markers identifying the different gene deletions. The table reports for each indicated genotype the total number of expected and found meiotic segregants, and among the latter the number of healthy, sick (i.e. producing a colony of small size) and inviable segregants. As previously reported [43], [49], [50], [65], [85], deletion of *ZDS1* and *ZDS2* decreases spore viability and increases sickness with variable penetrance, whereas deletion of *IGO1* and *IGO2* is well tolerated. B. Stationary phase cells of meiotic segregants with the indicated genotype that were classified as “healthy” in (A) were spotted on YEPD plates and incubated for 2 days at 25°C and 37°C. Deletion of *IGO1* and *IGO2* increases the temperature-sensitivity of healthy *zds1Δ zds2Δ* cells (similar results were obtained with several independent healthy meiotic segregants). C. Images of logarithmically growing cells with the indicated genotypes grown in YEPD at 25°C and shifted for 3 hours to 37°C.
(PDF)

Table S1 List of strains used in this study.
(PDF)
