# Supplementary figures and images for: Budding Yeast Greatwall and Endosulfines Control Activity and Spatial Regulation of PP2ACdc55 for Timely Mitotic Progression
Source: PLoS Genet. 2013 Jul 4;9(7):e1003575. doi: 10.1371/journal.pgen.1003575 (PMC3701715; doi:10.1371/journal.pgen.1003575)

**A**

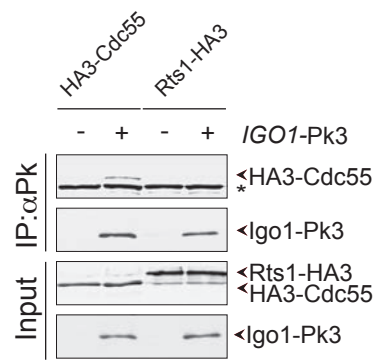

**B**

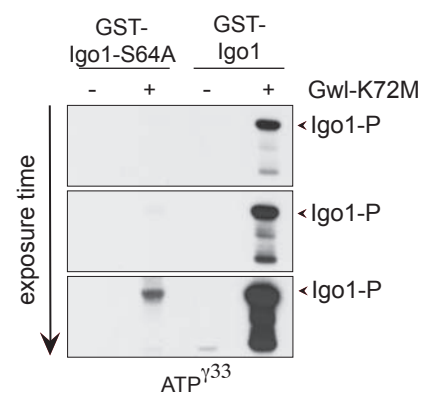

Supplement: Figure S1 — Igo1 interacts with the PP2A B subunit Cdc55 and not with its B′ subunit Rts1. A. Interaction between Igo1-Pk3 and either HA3-Cdc55 or Rts1-HA3 was assessed by immunoprecipitation with anti-Pk antibodies followed by western blot analysis with anti-HA and anti-Pk antibodies. B. Bacterially purified GST-Igo1 or GST-Igo1-S64A were phosphorylated in vitro by human Gwl using P32γATP. Phosphorylated Igo1 was visualized after SDS page and autoradiography. (PDF) [file pgen.1003575.s001.pdf]

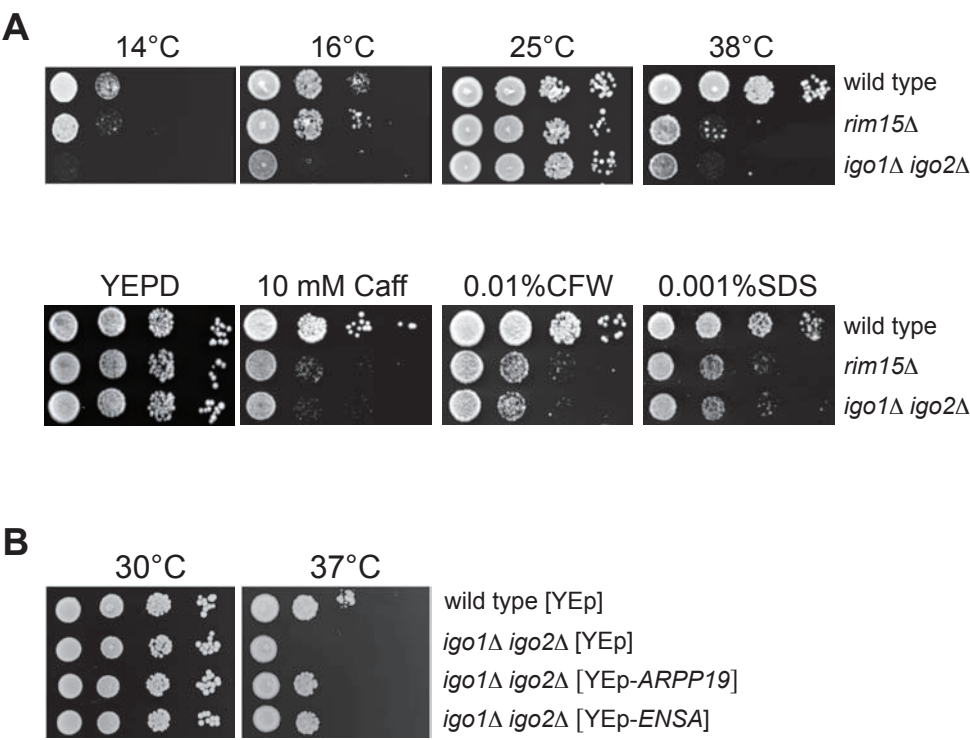

Supplement: Figure S2 — Cells lacking Rim15 or Igo1 and Igo2 are sensitive to different stress conditions and their temperature-sensitivity is suppressed by expression of human endosulfines. A. Serial dilutions of strains with the indicated genotypes were spotted on YEPD plates and incubated at the indicated temperatures (top panel) or were spotted on YEPD containing 10 mM caffeine (Caff) or 0.01% calcofluor white (CFW) or 0.001% SDS and incubated at 25°C (bottom panel). B. Wild type and igo1Δ igo2Δ cells carrying the empty vector (YEp) or a high copy number plasmid carrying human ARPP19 (YEp-ARPP19) or human ENSA (YEp-ENSA) under the control of the IGO1 promoter [40] were spotted on SD-Ura plates and incubated for two days at 37°C. (PDF) [file pgen.1003575.s002.pdf]

**A**

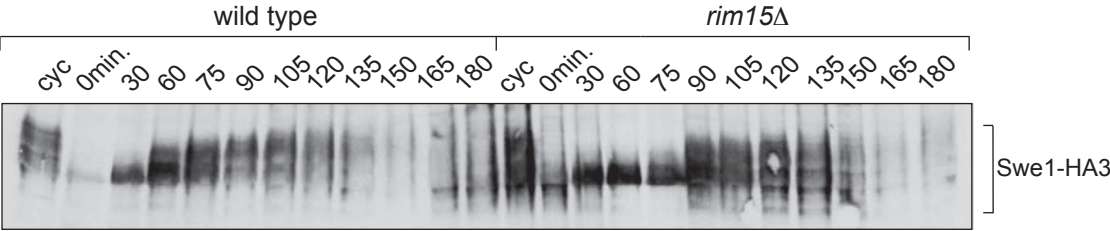

**B**

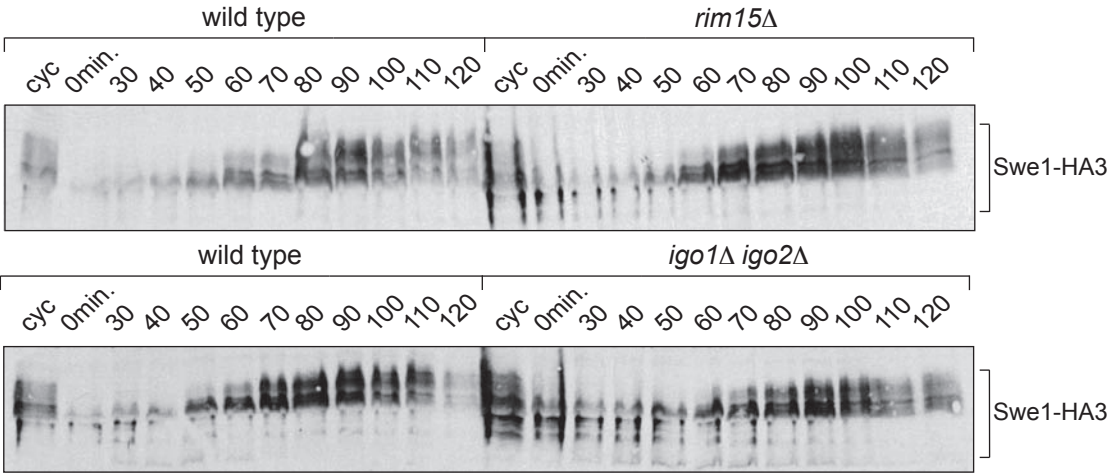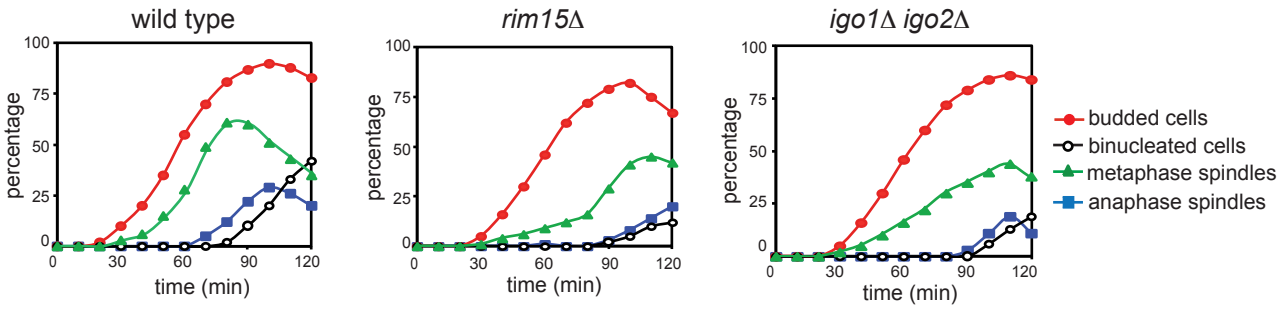

Supplement: Figure S3 — Deletion of RIM15 or IGO1 and IGO2 affects Swe1 hyperphosphorylation. Cycling (cyc) cultures of wild type, rim15Δ and igo1Δ igo2Δ cells expressing HA-tagged Swe1 (Swe1-HA3) were arrested in G1 by α-factor and released in fresh medium at 25°C in the presence of nocodazole (A) or at 38°C (B). At the indicated time points cells were collected for FACS analysis of DNA contents (not shown), kinetics of budding, spindle assembly/elongation and nuclear division (graphs), as well as to prepare TCA protein extracts for western blot analysis of Swe1-HA3 using anti-HA antibodies. Similar results were obtained in (A) for rim15Δ and igo1Δ igo2Δ cells (not shown). (PDF) [file pgen.1003575.s003.pdf]

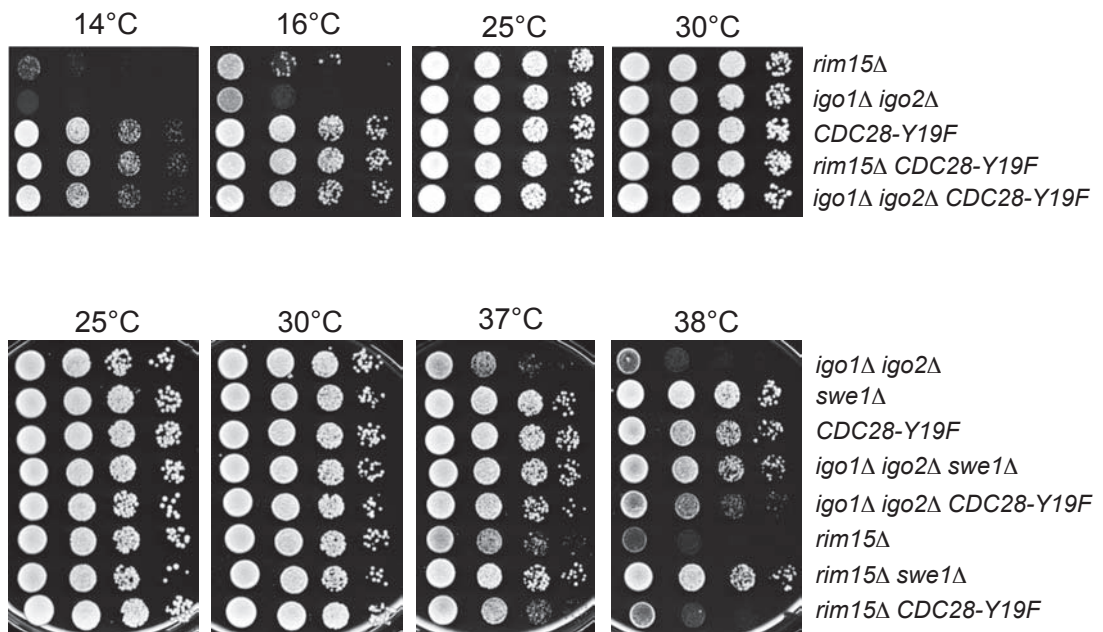

Supplement: Figure S4 — Expression of the non-phosphorylatable Cdc28-Y19F mutant protein suppresses the temperature-sensitive growth defects of rim15Δ and igo1Δ igo2Δ cells. Serial dilutions of strains with the indicated genotypes were spotted on YEPD plates and incubated for 48 hours at the indicated temperatures. (PDF) [file pgen.1003575.s004.pdf]
