# Supplementary material for: Budding Yeast Greatwall and Endosulfines Control Activity and Spatial Regulation of PP2ACdc55 for Timely Mitotic Progression
Source: PLoS Genet. 2013 Jul 4;9(7):e1003575. doi: 10.1371/journal.pgen.1003575 (PMC3701715; doi:10.1371/journal.pgen.1003575)

A

	EXPECTED	FOUND	HEALTHY	SICK	INVIABLE
igo1Δ igo2Δ	25,75	17	17	0	0
zds1Δ zds2Δ	25,75	24	8	11	5
zds1Δ zds2Δ igo1(2)Δ	51,5	47	29	13	5
zds1Δ zds2Δ igo1Δ igo2Δ	25,75	20	9	4	7

B

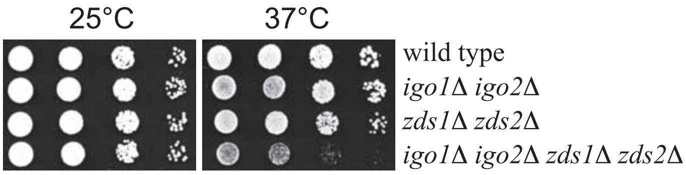

C

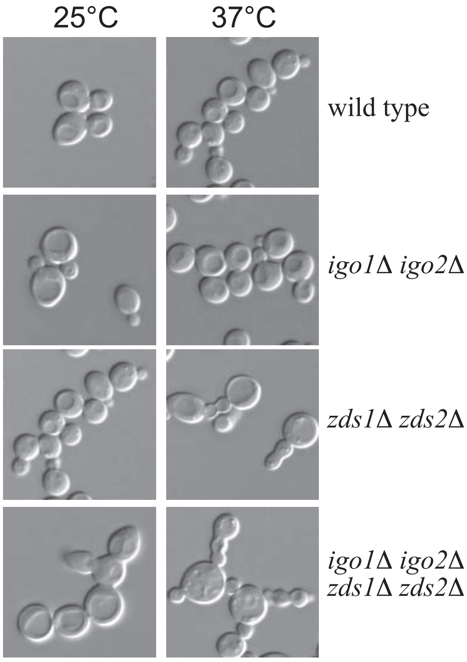

Supplement: Figure S5 — Deletion of IGO1 and IGO2 causes synthetic sickness to cells lacking Zds proteins. A. A heterozygous IGO1/igo1Δ IGO2/igo2Δ ZDS1/zds1Δ ZDS2/zds2Δ diploid strain was induced to sporulate and its meiotic segregants were analysed in 103 independent tetrads for the presence of genetic markers identifying the different gene deletions. The table reports for each indicated genotype the total number of expected and found meiotic segregants, and among the latter the number of healthy, sick (i.e. producing a colony of small size) and inviable segregants. As previously reported [43], [49], [50], [65], [85], deletion of ZDS1 and ZDS2 decreases spore viability and increases sickness with variable penetrance, whereas deletion of IGO1 and IGO2 is well tolerated. B. Stationary phase cells of meiotic segregants with the indicated genotype that were classified as “healthy” in (A) were spotted on YEPD plates and incubated for 2 days at 25°C and 37°C. Deletion of IGO1 and IGO2 increases the temperature-sensitivity of healthy zds1Δ zds2Δ cells (similar results were obtained with several independent healthy meiotic segregants). C. Images of logarithmically growing cells with the indicated genotypes grown in YEPD at 25°C and shifted for 3 hours to 37°C. (PDF) [file pgen.1003575.s005.pdf]
