# Supplementary material for: Budding Yeast Greatwall and Endosulfines Control Activity and Spatial Regulation of PP2ACdc55 for Timely Mitotic Progression
Source: PLoS Genet. 2013 Jul 4;9(7):e1003575. doi: 10.1371/journal.pgen.1003575 (PMC3701715; doi:10.1371/journal.pgen.1003575)
Supplement: Table S1 — List of strains used in this study. (PDF) [file pgen.1003575.s006.pdf]

Table S1. List of *Saccharomyces cerevisiae* strains used in this study

Name	Relevant genotype
ySP1370	*MATa, swe1::LEU2*
ySP2586	*MATa, mih1::LEU2*
ySP3427	*MATa, SWE1-HA3::URA3*
ySP3463	*MATalpha, HA3-CDC55*
ySP3645	*MATa, cdc55::KlTRP1*
ySP5652	*MATa, ura3::4X URA3::GAL1-CDC55,*
ySP6188	*MATa, cdc55::KanMX4*, *swe1::LEU2*
ySP8760	*MATa, rim15::natNT2*
ySP8785	*MATa, igo1::natNT2, igo2::KanMX4*
ySP8906	*MATa, igo1::natNT2, igo2::KanMX4, ura3::4X URA3::GAL1-CDC55*
ySP8929	*MATa, rim15::natNT2, ura3::4X URA3::GAL1-CDC55*
ySP9133	*MATa, RTS1-HA3::URA3*
ySP9207	*MATa, HA3-CDC55, IGO1-PK3::KlHIS3*
ySP9209	*MATa, RTS1-HA3::URA3, IGO1-PK3::KlHIS3*
ySP9258	*MATa, igo1::natNT2, igo2::KanMX4, swe1::LEU2*
ySP9341	*MATa, rim15::natNT2, HA3-CDC55, IGO1-PK3::KlHIS3*
ySP9350	*MATa, igo1::natNT2, igo2::KanMX4, [CEN-LEU2-IGO1- S64A-myc8]*
ySP9351	*MATa, igo1::natNT2, igo2::KanMX4, [CEN- LEU2-IGO1-myc8]*
ySP9440	*MATa, rim15::natNT2, [CEN- LEU2-IGO1-myc8]*
ySP9472	*MATa, igo1::natNT2, igo2::KanMX4, mih1::LEU2*
ySP9498	*MATa, igo1::natNT2, igo2::KanMX4*
ySP9511	*MATa, rim15::natNT2, mih1::LEU2*
ySP9520	*MATa, rim15::natNT2, swe1::LEU2*
ySP9650	*MATa, igo1::natNT2, igo2::KanMX4, HA3-CDC55*
ySP9655	*MATa, zds1::KlTRP1, zds2::HPHMX*
ySP9671	*MATa, igo1::natNT2, igo2::KanMX4, HA3-CDC55, [CEN-LEU2-IGO1- S64A-myc8]*
ySP9673	*MATa, igo1::natNT2, igo2::KanMX4, HA3-CDC55, [CEN-LEU2-IGO1-myc8]*
ySP9761	*MATa, igo1::natNT2, igo2::KanMX4, rim15::natNT2, HA3-CDC55*

ySP9794	*MATa, igo1::natNT2, igo2::KanMX4, [YEp-URA-ENSA]*
ySP9795	*MATa, igo1::natNT2, igo2::KanMX4, [YEp-URA-ARPP19]*
ySP9803	*MATa, igo1::natNT2, igo2::KanMX4, rim15::natNT2, HA3-CDC55, [CEN- LEU2-IGO1-myc8]*
ySP9834	*MATalpha, zds1::KlTRP1, zds2::HPHMX, HA3-CDC55, IGO1-PK3::KlHIS3*
ySP9835	*MATalpha, zds1::KlTRP1, zds2::HPHMX, HA3-CDC55*
ySP9855	*MATa, igo1::natNT2, igo2::KanMX4, rim15::natNT2, HA3-CDC55, [CEN- LEU2-IGO1-S64A-myc8]*
ySP9856	*MATa, igo1::natNT2, igo2::KanMX4, rim15::natNT2, HA3-CDC55, [CEN-LEU2]*
ySP9884	*MATa, ZDS1-PK6::KlHIS3, HA3-CDC55*
ySP9885	*MATalpha, igo1::natNT2, igo2::KanMX4, ZDS1-PK6:: KlHIS3, HA3-CDC55*
ySP10059	*MATa, igo1::natNT2, igo2::KanMX4, zds1::KlTRP1, zds2::HPHMX*
ySP10279	*MATa, igo1::natNT2, igo2::KanMX4, SWE1-HA3::URA3*
ySP10283	*MATa, rim15::natNT2, CDC28-Y19F::TRP1*
ySP10285	*MATa, igo1::natNT2, igo2::KanMX4, CDC28-Y19F::TRP1*
ySP10307	*MATa, MIH1-HA3::URA3*
ySP10308	*MATa, rim15::natNT2, MIH1-HA3::URA3*
ySP10309	*MATa, igo1::natNT2, igo2::KanMX4, MIH1-HA3::URA3*
ySP10310	*MATa, rim15::natNT2, HA3-CDC55*
ySP10311	*MATa, igo1::natNT2, igo2::KanMX4, swe1::LEU2, HA3-CDC55*
ySP10315	*MATa, rim15::natNT2, SWE1-HA3::URA3*
ySP10317	*MATa, cdc55::KlTRP1, MIH1-HA3::URA3*

Plasmids are indicated in brackets
